# Titanium Plasma-Sprayed Coatings on Polymers for Hard Tissue Applications

**DOI:** 10.3390/ma11122536

**Published:** 2018-12-13

**Authors:** Artur Wypych, Piotr Siwak, Daniel Andrzejewski, Jaroslaw Jakubowicz

**Affiliations:** 1 Institute of Materials Science and Engineering, Poznan University of Technology, Jana Pawla II 24, 61-138 Poznan, Poland; artur.wypych@put.poznan.pl; 2 Institute of Mechanical Technology, Poznan University of Technology, Piotrowo 3, 60-965 Poznan, Poland; piotr.siwak@put.poznan.pl; 3 Metal Forming Institute, Jana Pawla II 14, 61-139 Poznan, Poland; daniel.andrzejewski@inop.poznan.pl

**Keywords:** plasma spraying, Ti coating, polymers, biomaterials

## Abstract

The paper presents the results of titanium plasma spraying (TPS) on polymer substrates. Polyethylene (PE300), polyamide PA6, and fiber glass-reinforced polyamide (PA6.6-GF30) were used as substrates. The PE300 and PA6.6-GF30 substrates exhibited appropriate behavior during the TPS process, whereas the PA6 substrate did not “accept” Ti during plasma spraying, and the coating did not form. The TPS coatings exhibited low porosity and high homogeneity, and they had a typical multilayer structure composed of Ti and its oxides. The nanoindentation test showed good mechanical properties of the coatings and demonstrated a hardness and a Young’s modulus of approximately 400 HV and 200 GPa, respectively. The bending test confirmed the good adhesion of the titanium coatings to the polymer substrates. The Ti coatings did not fall off the substrate after its significant bending deformation.

## 1. Introduction

Metallization of polymer materials is a potentially good choice in terms of the improvement of their mechanical properties, elastic modulus, stiffness, hardness, and wear resistance. Elements made from polymer covered with metallic coatings are lighter than bulk metallic biomaterials and stronger than biopolymers without metallic coatings. The plasma spraying process is useful for the deposition of different types of coatings on biomaterials [1,2]. Plasma spraying of titanium powders (TPS—titanium plasma spraying) is commonly used for the enhancement of osseointegration through the formation of a rough, biocompatible surface [3]. In the process, the Ti powders are introduced into the stream of high-temperature plasma, melted, and deposited with high velocity on the substrate. Usually, the process is used for metallic substrates [4,5]. Recently, some attempts have been made to deposit Ti on polymer substrates, such as polyether ether ketone (PEEK) [6,7], although most of the attempts focused on the cold spraying process [8] at a significantly lower temperature than that of high-temperature plasma spraying. During plasma spraying, the Ti powder particles are liquid when flowing from the plasma gun nozzle onto the surface. However, their high velocity and energy, and their relatively large area of particles flattened during contact with the substrate, result in a great ability to dissipate energy. Hence, the substrate usually softens but does not melt.

The properties of many polymer substrates may be incompatible with the sprayed material. During plasma spraying, the substrate should have comparable thermal properties with the sprayed coating material, which would provide proper coating integrity with the substrate without cracks and delamination at the substrate–coating interphase.

The relatively poor knowledge related to the TPS process on polymer substrates and a very high application potential of the Ti coatings on polymers have motivated the authors to study this field. In this work, the authors show the results of the TPS coating formation on selected synthetic biopolymer substrates that may be potential candidate materials for hard tissue applications. In some applications, polymer materials with Ti coatings could provide sufficient strength at a low weight and hence could phase out metallic ones of greater weight.

## 2. Materials and Methods

In this work, titanium particles (average size 100 mesh = 149 µm, purity 99.7%; Sigma-Aldrich, St. Louis, MO, USA) were used for the plasma spraying process for coating preparation. The TPS coatings were made on polymer substrate rods (ϕ15 mm): high-density polyethylene PE300, polyamide PA6, and polyamide reinforced with 30% glass fibers (PA6.6-GF30) (Table 1).

In order to prepare the polymer surface to provide adhesion of the coating, abrasive blasting was applied. Owing to the softness of the polymer substrate, the energy of the abrasive Al_2_O_3_ particles (100 mesh, Alfa Aesar, Ward Hill, MA, USA) was reduced to prevent merging into the substrate. The particles were transported in air at a pressure of approximately 0.3 MPa. To generate the plasma stream, the AP-50 Plasma Spray System (Flame Spray Technologies, Duiven, The Netherlands) was used. The process parameters were automatically selected and controlled by programmable logic controllers (PLC) when the plasma generator operates at a current of up to 400 A. The JP-5000 plasma gun (Praxair Surface Technologies, Indianapolis, IN, USA) was used for the TPS process with a mixture of He (0.8 MPa) and H_2_ (0.15 MPa). The distance of the plasma gun to the surface was fixed at 480 mm. The diameter of the spraying field was 25 mm. The plasma spraying parameters were optimized for polymer substrates in our preliminary experiments (not shown here).

The structure analysis was performed using a PANalytical X-Ray diffractometer (Cu Kα radiation, Empyrean model, Royston, UK). The ICDD-JCPDS (International Centre for Diffraction Data-Joint Committee on Powder Diffraction Standards) crystallographic database was used for the phase identification. The microscopic observations were conducted using a GX51 optical microscope (Olympus, Tokyo, Japan) and Vega 5135 SEM (Tescan, Brno, Czech Republic). The coating porosity was estimated based on the SEM (Scanning Electron Microscope) images analyzed using Corel software (Corel Photo-Paint X5). The black spots in the coatings were qualified as the pores and the percentage porosity in the considered area was calculated.

The roughness was measured using an SRT-6210 roughness tester (Landtek, Guanghzou, China). In order to obtain the statistics to evaluate average roughness, three measurements of 2.5 mm scan length on different spots of the sample were performed. A Gaussian type filter was used for the profiles, and the cut-off length for the waviness-roughness separation was 0.25 mm. Uncertainties of the roughness measurements result from the accuracy of the testing device and are less than 10% of the indication.

The mechanical properties were measured using a Picodentor HM500 (Fischer, Windsor, CO, USA) nanoindentation tester at the indentation force load of 300 mN that was applied for 20 s. The load–displacement curves were recorded for the substrate and the coating. The Vickers Hardness (HV), the indentation modulus (E_IT_), the indentation creep (C_IT1_), the total mechanical work of indentation (W_t_), and the plastic deformation portion (N_plast_) were all measured.

The three-point bending test was performed using a 4483 mechanical testing machine (Instron, Norwood, MA, USA) with a constant crosshead speed of 4 mm/min. The distance between the supports was 50 mm and the bending mandrel was 10 mm in diameter. The bending was carried out up to a displacement of the bending mandrel by 19–20 mm, which corresponds to approximately 45°, or to the point when the sample breaks. The mechanical properties were measured for five samples of each series.

## 3. Results and Discussion

The titanium plasma spraying process applied to polymer rods resulted in a formation of relatively thick coatings of high roughness. The core material and its properties (Table 1) affected the deposition of the Ti particles, the formation of the coating, and its final properties.

**Figure 1:**
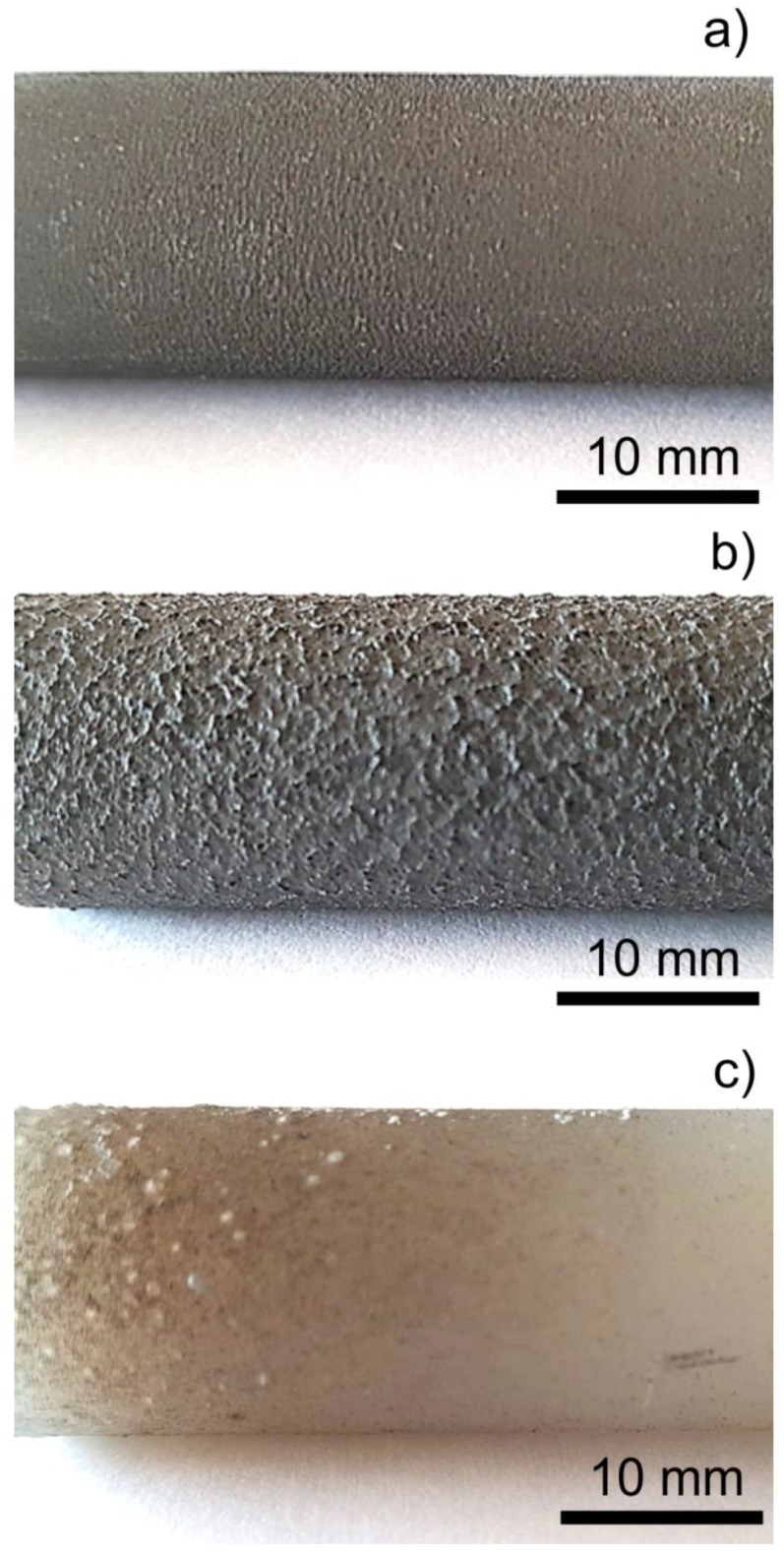
Titanium plasma spraying (TPS) coatings on the (**a**) PE300, (**b**) PA6.6-GF30, and (**c**) PA6 rods.

Among the three different substrate materials, the PA6 was found to be inadequate for the formation of the Ti coating (Figure 1). The surface of PA6 (Figure 1c) was incoherent with the sprayed Ti particles at the process setup, whereas for PE300 (Figure 1a) and PA6.6-GF30 (Figure 1b), the Ti coatings had sufficient adhesion, thickness, and roughness. The PA6 substrate material underwent surface transformation through significant softening and formed polymer blisters under the stream of melted Ti particles, which resulted in a failure to form the coating (Figure 1c). For both the PE300 and the PA6.6-GF30 substrate materials, the deposited Ti particles formed coatings.

The roughness of the Ti coatings was sufficiently high for the potential osseointegration process. The average values of the roughness parameters R_a_, R_z_, R_q_, and R_t_ for Ti deposited on PE300 were 25.5, 72.2, 26.1, and 72.9 µm, respectively, whereas for PA6.6-GF30, the roughness parameters R_a_, R_z_, R_q_, and R_t_ assumed the values of 25.4, 71.8, 22.7, and 77.0 µm, respectively. Given the macroscale topography of the coating, the more developed surface was created for PA6.6-GF30 in comparison to PE300. However, the roughness parameters, averaged from the given measurement area, were comparable for both samples. The roughness parameters R_a_, R_z_, R_q_, and R_t_ for the PA6 rod with the failed Ti coating assumed the values of 8.0, 22.6, 7.4, and 23.6 µm, respectively. For comparison, the typical average roughness R_a_ of the plasma-sprayed titanium coating is around 26 µm [9].

**Figure 2:**
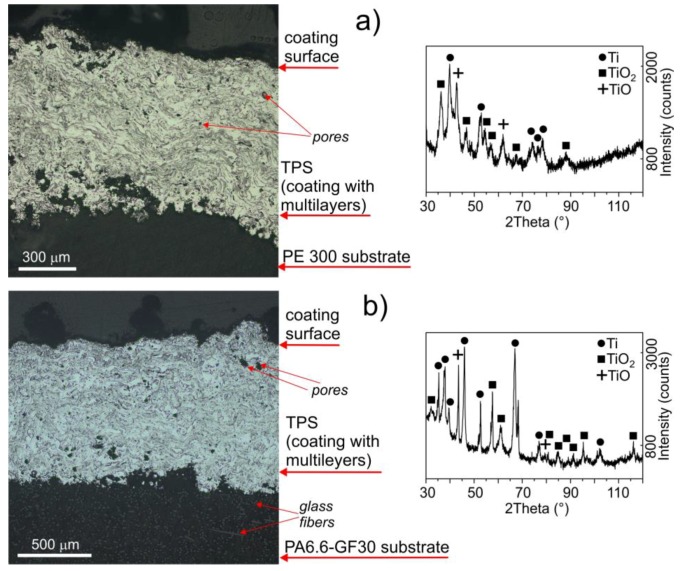
Microstructure as seen with an optical microscope and XRD spectra for the TPS coatings on (**a**) PE300 and (**b**) PA6.6-GF30.

The TPS coatings deposited on the polymer rods, i.e., PE300 and PA6.6-GF30, had a typical microstructure regardless of the substrate material used (Figure 2). Coatings deposited on metallic substrates, for example, Ti alloys, have a comparable microstructure and morphology [9]. The coatings are composed of multilayers because the plasma gun moves repeatedly along the surface, spraying the particles to obtain a coating of the desired thickness. The first sprayed Ti particles are mechanically embedded into the polymer substrate that is softened under high temperature. When the small liquid Ti particles hit the substrate, they undergo fast solidification and cooling, which does not increase the polymer substrate temperature beyond 80‒100 °C. This leads to a mechanical connection of the coating with the substrate. The Ti coatings described in this study had a uniform morphology with an internal porosity of <4.8% and <3.4% on PE300 and PA6.6-GF30, respectively. The porosity is heavily dependent on the TPS process optimization [10] and can reach up to 15% [9]. The thickness of the applied coatings fell into the range of 0.6 to 1.0 mm, which should be sufficiently high to provide good mechanical and physicochemical properties of the polymer elements for hard tissue application, for example, its coating integrity, its fixation with substrate, its continuity, and its density [7]. The typical thickness of plasma-sprayed titanium or hydroxyapatite coatings on Ti alloy substrates is in the approximate range of 0.2–0.6 mm [11,12]. Thicker coatings, however, provide better insulation of the substrate from the tissue [11]. Cold-sprayed metals on polymer substrates have a thickness of up to 1 mm [8]. The main phase in the coatings is pure Ti; however, oxides such as TiO_2_ and TiO are also present.

**Figure 3:**
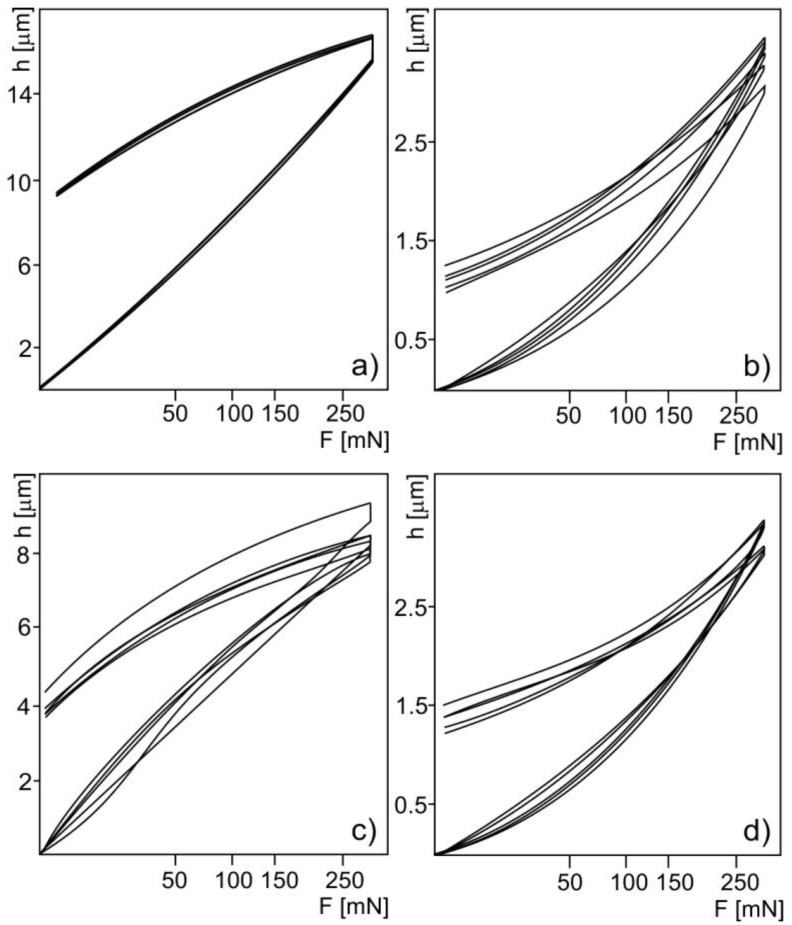
Load-displacement curves for (**a**) PE300 substrate, (**b**) TPS coating on PE300, (**c**) PA6.6-GF30 substrate, and (**d**) TPS coating on PA6.6-GF30.

**Figure 4:**
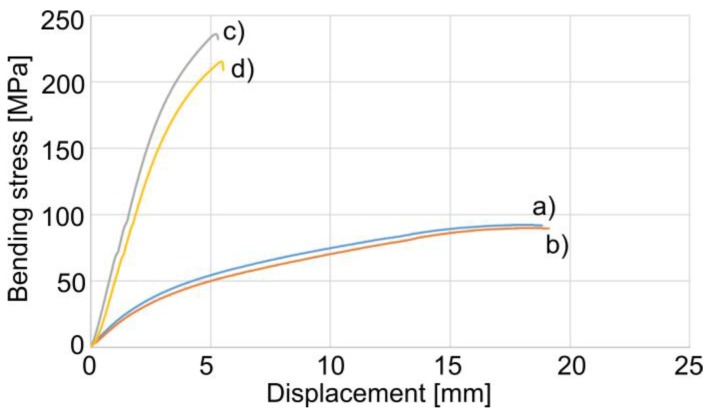
Bending test of the substrate polymer rods (**a**, **c**) and those with the TPS coating (**b**, **d**), (**a**, **b**) PE300, and (**c**, **d**) PA6.6-GF30.

The mechanical properties were measured by nanoindentation (Table 2, Figure 3) and bending tests (Figure 4). The nanoindentation was performed on polymer substrates, coatings, and bulk Ti for comparison. The load-displacement curves for the polyethylene substrate (Figure 3a) overlapped each other, exhibiting good material homogeneity. For the polyamide–glass fiber composite substrate (Figure 3c), the curves had a wide spread, which was a result of the two-phase polyamide–glass fiber substrate composition. The curves for the coatings (Figure 3b,d) had some spread that resulted from the interlayered morphology of the coatings, oxide distribution, and porosity. No delamination was observed at the diamond tip loading. The mechanical properties of the coatings were confirmed by the XRD analysis. The high hardness and Young’s modulus of the Ti coatings resulted from the presence of oxygen (TiO, TiO_2_), which significantly boosts the mechanical properties [13]. The mixture of oxides and pure Ti, as well as the multilayer morphology, acts against coatings delamination, as the layers and oxides facilitate plastic deformation through the slip mechanism, and brittle cracking.

The bendability of the PE300 type samples was higher in comparison to the PA6.6-GF30 ones (Figure 4). The PE300 samples did not crack at the displacement set on the machine (approximately 19 mm), whereas the PA6.6-GF30 samples, owing to the fiber glass reinforcement and thus greater stiffness, broke at the displacement of approximately 5.4 mm. The measured maximum bending stress at a given displacement for PE300 and PE300 with TPS was 92 MPa and 90 MPa, respectively. For PA6.6-GF30 and PA6.6-GF30 with TPS, the maximum bending stress (strength) was 236 MPa and 215 MPa, respectively. The samples with the TPS coatings exhibit a lower bending stress in comparison to the substrate rods without the coatings. This behavior correlated with the coating properties. During the bending test, the TPS coating deformed, cracked, and easily slid on the machine test supports—better than the polymer substrate—and the coating was pressed into the polymer substrate by the mandrel and the supports of the testing machine. The coatings bent together with the polymer substrate without delaminating. Additionally, the relatively low thickness and the multilayer morphology of the coatings resulted in lower stress during the bending test for the TPS samples.

**Figure 5:**
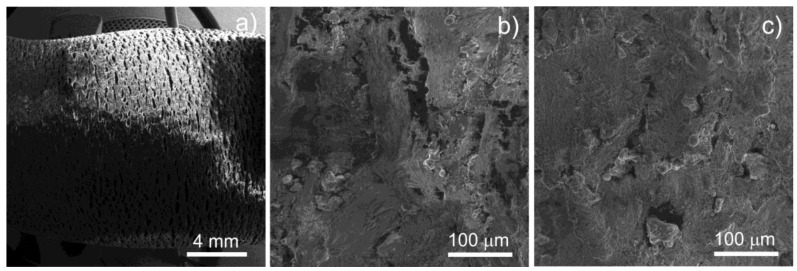
SEM images of the TPS coating on the PE300 substrate after the bending test; (**a**) low magnification sample view, (**b**) the extended side of the sample, and (**c**) the compressed side of the sample.

The Ti coatings sprayed on the polymer substrate exhibited good adhesion qualities. In the bending tests, the coatings cracked under a high level of substrate deformation. However, they did not delaminate but adhered to the polymer surface (Figure 5). Due to the bending behavior, one side of the sample was compressed (the part of the sample affected by the mandrel) and the opposite side (from the supports side) was extended. The extended side revealed more cracks (Figure 5a,b) compared to the compressed side (Figure 5c). In both cases, however, the coatings did not fall off the substrate. 

## 4. Conclusions

In this work, the authors investigated the method of titanium plasma spraying on polymer substrates. The Ti coatings exhibit a typical multilayer morphology of low porosity and good adhesion to polyethylene and glass fiber-reinforced polyamide. For pure polyamide, the TPS process was unsuccessful. The coatings are composed of Ti and its oxides, which resulted in the coatings’ greater hardness of approximately 400 HV and a Young’s modulus of approximately 200 GPa. The coating did not improve the bending strength, yet the bending tests have shown good adhesion of the coatings.

The preliminary results shown in this paper are an introduction to stimulate further research into the TPS process with polymer substrates with a view to its optimization and practical application.

## Figures and Tables

**Table 1:** Properties of the polymer substrates and the coating material.

Properties	PE300	PA6	PA6.6-GF30	Ti
Density (g/cm^3^)	0.96	1.14	1.36	4.51
Yield strength (MPa)	25	76	180	280
Tensile strength (MPa)	36	79	210	350
Elongation (%)	50	50	5	20
Modulus of elasticity (GPa)	1.0	3.3	7.0	105
Hardness (MPa)	45	155	200	970
Thermal conductivity (W/mK)	0.41	0.28	0.23	17
Coefficient of linear thermal expansion (×10^−6^ K^−1^)	200	90	30	8.4

**Table 2:** Mechanical properties of the investigated polymer substrates, Ti coatings, and bulk Ti.

Material	HV	E_IT_ (GPa)	W_t_ (µJ)	N_plast_ (%)	C_IT_ (%)
PE300 substrate	5.43 ± 0.04	1.28 ± 0.02	1.67 ± 0.02	65.0 ± 0.3	6.8 ± 0.2
Ti coating (on PE300 substrate)	396.7 ± 34.5	204.6 ± 27.3	0.38 ± 0.06	30.0 ± 5.8	5.6 ± 1.0
PA6.6-GF30 substrate	20.3 ± 2.1	4.82 ± 0.59	0.74 ± 0.07	55.3 ± 1.3	4.9 ± 0.8
Ti coating (on PA6.6-GF30 substrate)	420.3 ± 39.2	214.4 ± 21.5	0.39 ± 0.07	32.0 ± 4.6	5.1 ± 0.8
Bulk Ti (for comparison)	189.3 ± 11.7	115.2 ± 3.1	0.28 ± 0.03	86.2 ± 1.2	3.3 ± 0.3
